# Cytonuclear diversity and shared mitochondrial haplotypes among *Daphnia galeata* populations separated by seven thousand kilometres

**DOI:** 10.1186/s12862-018-1256-4

**Published:** 2018-09-03

**Authors:** Mingbo Yin, Xiaoyu Wang, Xiaolin Ma, Sabine Gießler, Adam Petrusek, Johanna Griebel, Wei Hu, Justyna Wolinska

**Affiliations:** 1 MOE Key Laboratory for Biodiversity Science and Ecological Engineering, School of Life Science, Fudan University, Songhu Road, Shanghai, 2005 China; 2 Department Biologie II, Aquatic Evolutionary Ecology, Ludwig-Maximilians-Universität, Großhaderner Str. 2, 82152 Planegg-, Martinsried, Germany; 3 Faculty of Science, Department of Ecology, Charles University, Viničná 7, 12844 Prague, Czechia; 4 Leibniz-Institute of Freshwater Ecology and Inland Fisheries, Department of Ecosystem Research, Mueggelseedamm 301, 12587 Berlin, Germany; 5 Department of Biology, Chemistry, Pharmacy, Institute of Biology, Freie Universität Berlin, Konigin-Luise-Str. 1-3, 14195 Berlin, Germany

**Keywords:** Cyclical parthenogenesis, Cladocera, Genetic variation, Microsatellites, 12S rRNA, Population structure

## Abstract

**Background:**

The zooplanktonic cladocerans *Daphnia*, present in a wide range of water bodies, are an important component of freshwater ecosystems. In contrast to their high dispersal capacity through diapausing eggs carried by waterfowl, *Daphnia* often exhibit strong population genetic differentiation. Here, to test for common patterns in the population genetic structure of a widespread Holarctic species, *D. galeata*, we genotyped two sets of populations collected from geographically distant areas: across 13 lakes in Eastern China and 14 lakes in Central Europe. The majority of these populations were genotyped at two types of markers: a mitochondrial gene (for 12S rRNA) and 15 nuclear microsatellite loci.

**Results:**

Mitochondrial DNA demonstrated relatively shallow divergence within *D. galeata*, with distinct haplotype compositions in the two study regions but one widely distributed haplotype shared between several of the Chinese as well as European populations. At microsatellite markers, clear separation was observed at both large (between China and Europe) and small (within Europe) geographical scales, as demonstrated by Factorial Correspondence Analyses, Bayesian assignment and a clustering method based on genetic distances. Genetic diversity was comparable between the sets of Chinese and European *D. galeata* populations for both types of markers. Interestingly, we observed a significant association between genetic distance and geographical distance for *D. galeata* populations in China but not in Europe.

**Conclusions:**

Our results indicate relatively recent spread of *D. galeata* across wide expanses of the Palaearctic, with one mtDNA lineage of *D. galeata* successfully establishing over large distances. Despite a clear differentiation of Chinese and European *D. galeata* at a nuclear level, the pattern of genetic variation is nevertheless similar between both regions. Overall, our findings provide insights into the genetic population structure of a cladoceran species with extremely wide geographical range.

**Electronic supplementary material:**

The online version of this article (10.1186/s12862-018-1256-4) contains supplementary material, which is available to authorized users.

## Background

A major challenge in our understanding of biodiversity is why certain species have a wide distribution, spanning several biogeographical regions, while others have much more restricted ranges or are endemic. The cosmopolitanism view [1] holds that species with large population sizes and strong dispersal abilities maintain genetic homogeneity across widely dispersed geographical regions. For example, many freshwater invertebrate species were considered to be cosmopolitan. This conclusion was based on observed morphological similarities of specimens inhabiting different continents and interpreted as a result of efficient dispersal mechanisms (e.g. [2]). However, the classical view of cosmopolitanism has been challenged by extensive genetic studies. Specifically, despite the morphological similarities across broad geographical ranges, many freshwater zooplankton species, including rotifers and cladocerans, have been shown to display strong genetic divergence not only at a global scale, but even at regional levels (e.g. [3, 4]). At present, it is possible to detect even weak differentiation of population structure within species, because of the wide availability of sufficiently variable multi-locus nuclear and mitochondrial DNA (mtDNA) markers. Besides tracing maternal lines via mtDNA, local adaptation processes can be examined by analysis of nuclear polymorphism (e.g. [5–7]).

Here, we focus on a common and very widespread freshwater zooplankton species, the cladoceran *Daphnia galeata* G. O. Sars, 1863, to improve our understanding of its dispersal and genetic diversity over small and large geographic scales. *Daphnia* (Anomopoda: Daphniidae) are important components of freshwater ecosystems, being grazers of phytoplankton as well as a key prey item of planktivorous fish [8]. *Daphnia* are cyclical parthenogens; females mostly reproduce clonally by giving birth to parthenogenetic daughters. In unfavourable environments, however, *Daphnia* switch to the production of males and sexual eggs which require fertilization, and then enter diapause in a gastrula stage [9]. *Daphnia galeata*, a member of the *D. longispina* complex (taxonomy revised in: [10]), has a wide Holarctic distribution [11], being common in freshwater lakes in Europe, North America and Asia (e.g. [11–16]). This broad geographical range of *D. galeata* apparently results from long-distance passive dispersal of sexually produced diapausing eggs, which can be carried among water bodies by waterfowl (e.g. [17, 18]). Surprisingly, despite the high dispersal capacity of anomopod cladocerans, substantial genetic differentiation has been detected among studied populations, across different *Daphnia* species (e.g. [5, 6]).

Ishida and Taylor [12] surveyed *D. galeata* populations across Holarctic continents, using both a mitochondrial DNA (mtDNA) marker and a conservative nuclear marker (i.e. HSP90). One major mtDNA type of *D. galeata* was detected throughout the Holarctic with some haplotypes shared over long distances, even between Europe and North America. At the nuclear marker level, however, New and Old World *D. galeata* were clearly differentiated [12]. Moreover, there was a mismatch between the patterns observed at these markers: five nuclear but only four mitochondrial phylogroups were detected [12], which was explained by nuclear introgression from *D. dentifera* to *D. galeata* in the New World. However, populations from China, where *D. galeata* is a very common zooplankter [15], were not included in that previous survey, in which continental Asia was represented only by Siberian populations. Our recent study of the biogeography and diversity of *D. galeata* across China revealed low mtDNA variation; a common *D. galeata* haplotype (as resolved by sequencing of 12S rRNA and cytochrome c oxidase subunit I) was widespread across China, Japan, Siberia and the Western Palaearctic [15]. We also detected many rare local mtDNA haplotypes in China, suggesting either that some time has passed since initial colonization, along with subsequent evolutionary change, or that multiple introductions occurred [15]. In contrast, nuclear differentiation based on variation of multiple microsatellite loci supported clear spatial differentiation among Chinese *D. galeata* populations even on a relatively small geographical scale (within 30 km, [19]).

The aim of the present study was to compare patterns in the genetic population structure of the most widespread species of the *D. longispina* complex, *D. galeata* [20, 21], between two distant areas, and to identify common or potentially distinct features. We compared the genotypic diversity of two sets of *D. galeata* populations (from Eastern China and Central Europe) using two types of markers, a mitochondrial gene (12S rDNA region) and 15 nuclear microsatellite loci. In the case of mtDNA, we expected pronounced divergence of *D. galeata* between the two study regions, but low variation within them, as mitochondrial DNA usually has low resolution to detect subtle within-species population differentiation (e.g. [22]). When applying microsatellite markers, we expected to observe significant variation among populations of *D. galeata*, both within and between remote regions. Genetic diversity in this instance is derived from a high number of fast evolving polymorphic loci and, because of their codominant inheritance, discrimination among populations at high resolution is possible [23].

## Methods

### Daphnia *specimen collection*

**Fig. 1:**
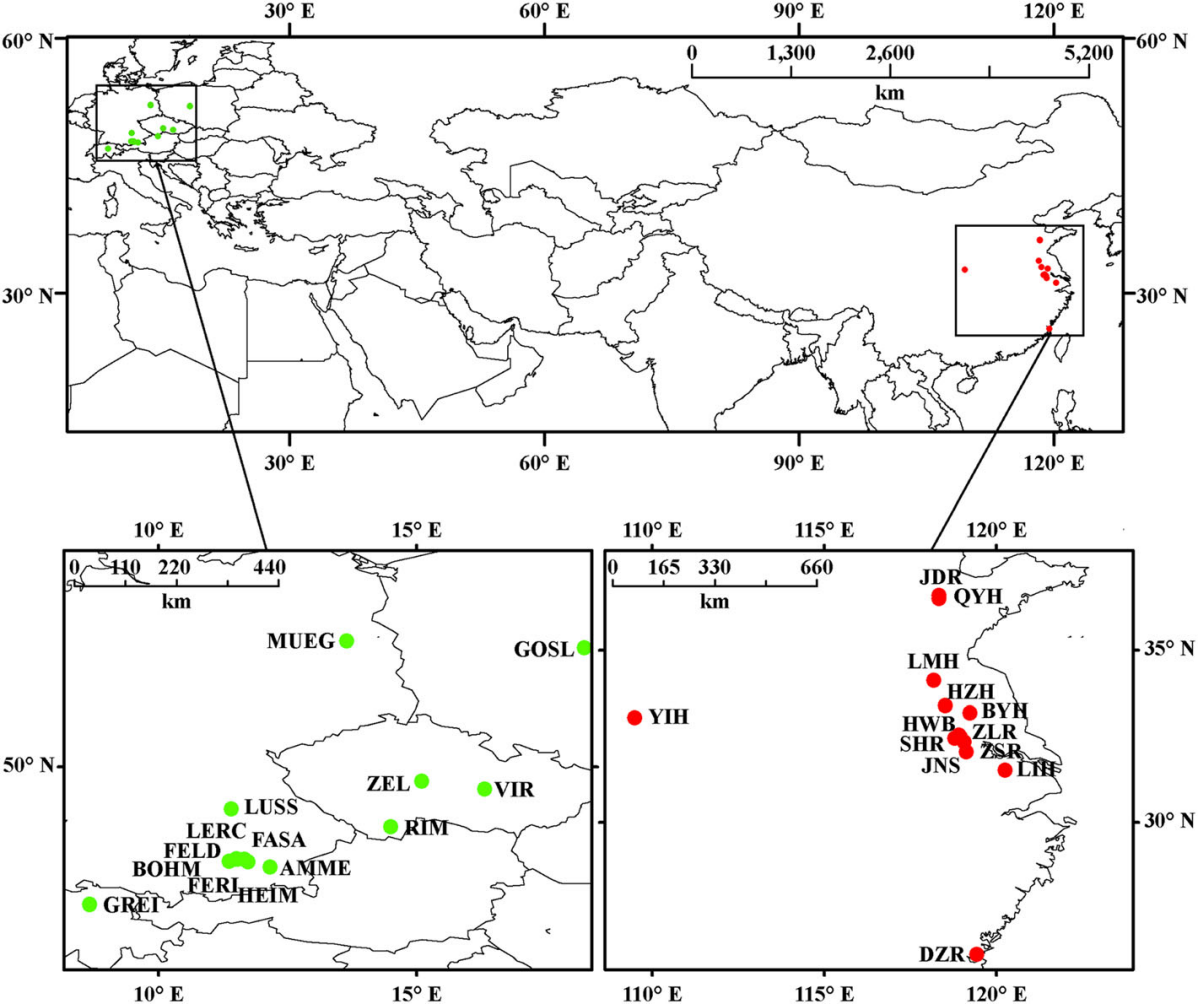
Location of *D. galeata* samples from Eastern China (red dots) and Central Europe (green dots)

Thirteen Chinese and fourteen European *Daphnia galeata* localities were analysed in this study (Fig. 1). All Chinese lakes are located in the eastern plain (lakes elsewhere in China are mostly inhabited by another species from the *D. longispina* complex, *D. dentifera* [15]). Among European localities, nine are located in Germany, one in Poland, one in Switzerland and three in Czechia (Table 1). The largest distance among lakes within each region was 1228 km in China, and 884 km in Europe; European and Chinese groups of lakes were over 7000 km apart. Zooplankton samples were collected with a 125-μm plankton net hauled through the whole water column at two to three different sites per lake (for sampling years and seasons see Table 1). Samples from different locations within lakes were pooled and preserved in 95% ethanol.

**Table 1:** Genetic diversity of *Daphnia galeata* populations from Eastern China and Central Europe, based on 15 microsatellite loci and a mitochondrial gene (for 12S rRNA)

	Locality (abbreviation), habitat type, country	Sampling period	Latitude, longitude	Microsatellites					12S				
				N_mic_	N_MLG_	MLG	R	*A*_*r*_	N_seq_	N_hapl_	*h*	*π*	haplotypes (N_ident_)
Europe	Ammersee (AMME)^a^, natural lake, DE	Autumn 2008	48°02′, 11°42′	12	12	11	0.91	2.57	n.a.				
	Böhmerweiher (BOHM)^c^, small flooded gravel pit, DE	Spring 2008	48°10′, 11°22′	n.a.					13	3	0.29	0.00067	CES (11), BOHMa (1), BOHMb (1)
	Fasanariesee (FASA), small flooded gravel pit, DE	Spring 2011	48°12′, 11°31′	43	41	17	0.40	2.30	10	1	0	0	CES (10)
	Feldmochinger See (FELD), small flooded gravel pit, DE	Spring 2009	48°07′, 11°18′	46	44	33	0.74	2.29	10	1	0	0	CES (10)
	Feringasee (FERI)^a,c^, small flooded gravel pit, DE	Spring 2009	48°07′, 11°24′	32	31	31	1	2.80	19	3	0.20	0.00071	ESa (17), FERIa (1), FERIb (1)
	Greifensee (GREI), natural lake, CH	Spring 2002	47°20′, 08°40′	36	35	21	0.59	3.49	10	2	0.53	0.00232	CES (6), ESb (4)
	Goslawskie (GOSL), natural lake, PL	Spring 2014	52°18′, 18°15′	43	36	31	0.86	4.25	10	2	0.36	0.00232	ESb (2), GOSLa (8)
	Heimstettener See (HEIM)^a^, small flooded gravel pit, DE	Spring 2008	48°06′, 11°26′	46	43	43	1	3.06	n.a.				
	Lerchenauer See (LERC), small flooded gravel pit, DE	Spring 2011	48°12′, 11°32′	45	44	31	0.70	3.07	10	1	0	0	CES (10)
	Lußsee (LUSS), small flooded gravel pit, DE	Spring 2008	49°12′, 11°25′	n.a.					12	2	0.17	0.00037	CES (11), LUSSa (1)
	Mueggelsee (MUEG), natural lake, DE	Spring 2016	52°26′, 13°38′	46	46	32	0.69	3.74	10	3	0.51	0.00385	ESa (7), ESb (2), MUGGa (1)
	Římov (RIM), man-made reservoir, CZ	Autumn 2009	48°50′, 14°30′	46	46	31	0.67	3.40	n.a.				
	Vír (VIR), man-made reservoir, CZ	Autumn 2009	49°34′, 16°19′	46	16	15	0.93	3.71	10	3	0.38	0.00174	CES (8), ESa (1), ESb (1)
	Želivka (ZEL), man-made reservoir, CZ	Summer 2009	49°43′, 15°06′	42	35	31	0.88	4.11	10	3	0.51	0.00358	CES (1), ESa (2), ESb (7)
China	Baoying Hu (BYH)^b,d^, natural lake	Spring 2012	33°06′, 119°08′	39	34	34	1	3.75	10	2	0.20	0.00218	HS1 (9), CES (1)
	Hewangba Reservoir (HWB)^b^, man-made reservoir	Spring 2013	32°32′, 118°50′	40	35	17	0.47	2.37	10	3	0.51	0.00121	HS1 (7), HWBa (2), HWBb (1)
	Hung-tse Lake (HZH)^b,d^, natural lake	Spring 2012	33°13′, 118°18′	44	40	29	0.72	3.08	10	1	0	0	HS1 (10)
	Jinniushan Reservoir (JNS)^b^, man-made reservoir	Spring 2013	32°28′, 118°57′	42	37	18	0.47	2.24	10	2	0.53	0.00116	HS1 (4), HS4 (6)
	Luoma Hu (LMH)^b,d^, natural lake	Autumn 2012	34°07′, 118°11′	43	33	33	1	3.17	10	2	0.53	0.00116	HS1 (6), HS4 (4)
	Shanhu Reservoir (SHR)^b^, man-made reservoir	Spring 2013	32°26′, 118°47′	22	17	17	1	2.50	10	1	0	0	HS1 (10)
	Zaolin Reservoir (ZLR)^b^, man-made reservoir	Spring 2013	32°20′, 119°04′	42	34	31	0.91	2.50	10	1	0	0	HS1 (10)
	Zhongshan Reservoir (ZSR)^b,d^, man-made reservoir	Autumn 2012	31°37′, 119°04′	41	34	22	0.64	2.30	10	1	0	0	HS1 (10)
	Li Hu (LIH), natural lake	Spring 2012	31°19′, 120°09′	40	34	34	1	3.73	10	5	0.84	0.00741	HS1 (3), CES (3), LIHa (1), LIHb (1), LIHc (2)
	Qinyun Hu (QYH)^d^, man-made reservoir	Spring 2012	35°54′, 117°48′	37	33	25	0.75	2.65	10	2	0.36	0.00387	HS1 (2), CES (8)
	Jingdong Reservoir (JDR)^d^, man-made reservoir	Spring 2012	35°57′, 117°48′	45	24	11	0.43	2.20	10	1	0	0	HS1 (10)
	Ying Hu (YIH)^d^, man-made reservoir	Autumn 2012	32°37′, 108°54′	44	26	21	0.80	3.06	10	3	0.62	0.00465	HS1 (6), HS4 (2), CES (2)
	Dongzhang Reservoir (DZR), man-made reservoir	Spring 2013	25°42′, 119°16′	42	22	13	0.57	1.34	10	1	0	0	HS1 (10)

Microsatellite data published in: ^a^ [24], ^b^ [19]. 12S data published in: ^c^ [42], ^d^ [15]. *N*_*mic*_ number of genotyped individuals, *N*_*MLG*_ number of genotyped individuals with a complete multilocus genotype (MLG), *MLG* number of detected multilocus genotypes, *R* relative clonal richness, *A*_*r*_ allele richness, *n.a.* data not available, *N*_*seq*_ number of sequenced individuals, *N*_*hapl*_ number of haplotypes per lake, *h* haplotype diversity per lake, *π* nucleotide diversity per lake, *N*_*ident*_ a number in brackets indicates number of individuals possessing the identical sequence. Haplotype ID: CES: haplotype shared by China and Europe; ES: haplotypes shared among European populations; HS: haplotypes shared among Chinese populations (IDs are consistent with [15]). Countries are indicated by two-letter ISO codes in the second column

### Nuclear DNA (microsatellites)

#### Genotyping

Thirteen Chinese and twelve European *D. galeata* populations were processed for analyses of microsatellites. The individual DNA extractions followed the protocol reported in [24]. Fifteen microsatellite loci [25] were amplified in two multiplex polymerase chain reactions [24]. The PCR products were then analysed on an ABI PRISM 3730 capillary sequencer, using a LIZ 500 labelled size standard. For each run, a reference *D. galeata* genotype (G100 from Europe) was used as a positive control to adjust for small differences in fragment length of identical alleles among runs. Genotypes were scored using GeneMapper version 3.7 (Applied Biosystems), and the alleles at each locus were defined by their fragment length (in base pairs). About 40 adult *D. galeata* females were genotyped from each Chinese population (except for SHR: 22 individuals, Table 1; 521 individuals in total). In Europe, *D. galeata* often coexists with other species from the *D. longispina* complex (e.g. [14, 24]). Recurrent hybridization has been reported [26–28] which requires careful multi-locus species identification [24, 29, 30]. Thus, only true *D. galeata* specimens (based on Bayesian analyses of microsatellite data and comparison of obtained results with reference species [19, 24]) were included in the analyses of European populations (about 40 individuals per population, except for AMME: 12 individuals, Table 1; 483 individuals in total). In addition to 14 newly sampled and genotyped populations, microsatellite analyses included data that have already been published from eight Chinese and three European lakes [19, 24] (see Table 1).

#### Verification of taxonomic assignment of Chinese and European *D. galeata*

 We applied Factorial Correspondence Analysis (FCA) on all 25 *D. galeata* populations, with similar settings to those previously used in [19]. Forty-nine well-defined genotypes, representing three species of the *D. longispina* complex (*D. galeata*, *D. longispina* and *D. cucullata*) and their interspecific hybrids, were included as reference taxa in FCA (the same reference genotypes were used in [24]). Then, NewHybrids [31] was applied to double-check if all FCA-identified individuals were pure *D. galeata*. Since NewHybrids allows the discrimination of only two species and their interspecific hybrids, the relevant species pair combinations (*galeata* and *longispina*; *galeata* and *cucullata*) were analysed separately. New Hybrids then assigned the newly analysed individuals together with reference individuals into six possible classes (i.e. two parental species and four hybrid classes: F1, F2 hybrids and both backcrosses). The analysis was run for 10^6^ iterations after a burn-in of length 10^6^, and the probability threshold for taxon identification was set to 90%. Only individuals with pure *D. galeata* assignments were used further.

#### Population structure and diversity

To explore the genetic relationship of *D. galeata* populations between and within regions, a similar FCA was run as described above, but without the reference genotypes. In order to identify genetically homogenous groups across all 25 *D. galeata* populations, a Bayesian algorithm was applied in STRUCTURE V2.3.4 [32], assuming the existence of *K* groups. For each tested value of *K* (i.e. 1 to 25), ten independent runs were performed and, for each run, 10^5^ iterations were carried out after a burn-in period of 10^5^ iterations. The most likely *K* was determined by the distribution of *ΔK*, following the methods of Evanno et al. [33]. Then, for each pre-defined population (i.e. lake), the proportion of individuals classified as belonging to each genetic group was calculated. Two similar runs in STRUCTURE were performed as described above to check for genetically homogenous groups within regions across 13 Chinese (*K*: 1 to 13) and 12 European (*K*: 1 to 12) *D. galeata* populations, respectively. Next, the genetic similarity among populations from different regions was estimated by constructing an Unweighted Pair-Group Method with Arithmetic Mean (UPGMA) dendrogram based on Nei’s genetic distances [34], as calculated in MEGA 5 [35]. A hierarchical analysis of molecular variance (AMOVA) was performed in Arlequin 3.11 [36] to partition the genetic variance among *D. galeata* populations into the following components: 1) between regions (i.e. Eastern China and Central Europe), 2) among populations within regions, and 3) among individuals within populations. To estimate genetic differentiation among *D. galeata* populations for each region, the fixation index *F*_*ST*_ was calculated in Arlequin 3.11 with 10^4^ permutations. Finally, the correlation between pairwise geographical distance and pairwise *F*_*ST*_ was computed using a Mantel test (10^4^ permutations, in Isolation by Distance Web Service, version 3.15 [37]). The level of genetic diversity in each *D. galeata* population was evaluated by calculating the allelic richness (*A*_*r*_), in FSTAT v.2.9.3.2 [38]. *D. galeata* from VIR and ZEL (for abbreviations of lakes see Table 1) could not be amplified at locus SwiD15, and this locus was removed from the analysis of allelic richness. Relative clonal richness (R) was calculated for each population as R = (G-1) / (N-1), where G is the number of genotypes and N indicates sample size [39]. In the analysis of clonal richness, only individuals characterized at all microsatellite loci were considered (individuals with missing data at locus SwiD15 from VIR and ZEL were also included); i.e. 832 of 1004 genotyped *D. galeata* individuals, see Table 1. Comparisons of genetic diversity indices between regions (i.e. Chinese vs. European populations) were made using Student’s *t* test. Data were tested for confirmation of a normal distribution by a Kolmogorov-Smirnov test, and deviating variables were transformed using the Rankit function [40].

### Mitochondrial DNA (12S gene)

#### Amplification and sequencing

Thirteen Chinese and eleven European populations were processed for analyses of mtDNA (130 Chinese and 124 European specimens in total, Table 1). DNA was extracted by the proteinase K method [41]. A 544 bp long segment of the small ribosomal subunit (12S rRNA) was sequenced [15]. The 12S sequence data from eight out of the thirteen studied Chinese lakes have been published elsewhere [15] as part of a geographical survey of *Daphnia* communities in China (see Table 1). Additional individuals were genotyped for each of these eight lakes, resulting in exactly ten analysed individuals per Chinese population. In the case of European lakes, 10 to 19 individuals were sequenced per population. Data from two out of the eleven studied European lakes have been published ([42]; see Table 1). Although most individuals in these two populations (BOHM and FERI) belonged to F1 *D. galeata* × *D. longispina* hybrids (as identified by 15 microsatellite loci; see [42]), they are included in the present study because their mtDNA sequences represented *D. galeata* maternal lineages. All newly obtained 12S rRNA sequences were submitted to GenBank under accession numbers MH052570-MH052577.

#### Population diversity and phylogenetic analyses

Haplotype diversity (*h*) and nucleotide diversity (*π*) for the 12S sequences were calculated in DnaSP Version 5 [43]. Then, genetic diversity indices were compared between Chinese and European populations with Student’s *t* test (data were transformed if necessary, see above). The unique haplotypes were identified in Arlequin, and then aligned together with 12S reference sequences of the *D. longispina* complex, including *D. galeata* from Asia (Japan) and Europe (see Additional file 1: Table S1), using Clustal W [35]. A phylogenetic tree was constructed by applying the Bayesian method in MrBayes 3.2, considering the estimated best-fit substitution models [44]. *Daphnia hrbaceki*, a member of the *D. curvirostris* complex genetically close to the *D. longispina* complex [45], was used as an outgroup. To estimate 12S mtDNA gene genealogies, a network analysis of *D. galeata* haplotypes was performed using Haploviewer [46], based on Bayesian algorithms. Finally, an AMOVA test comparable to that applied to microsatellite data (see above) was run on 12S mtDNA sequence data.

## Results

### Nuclear DNA (microsatellites)

**Fig. 2:**
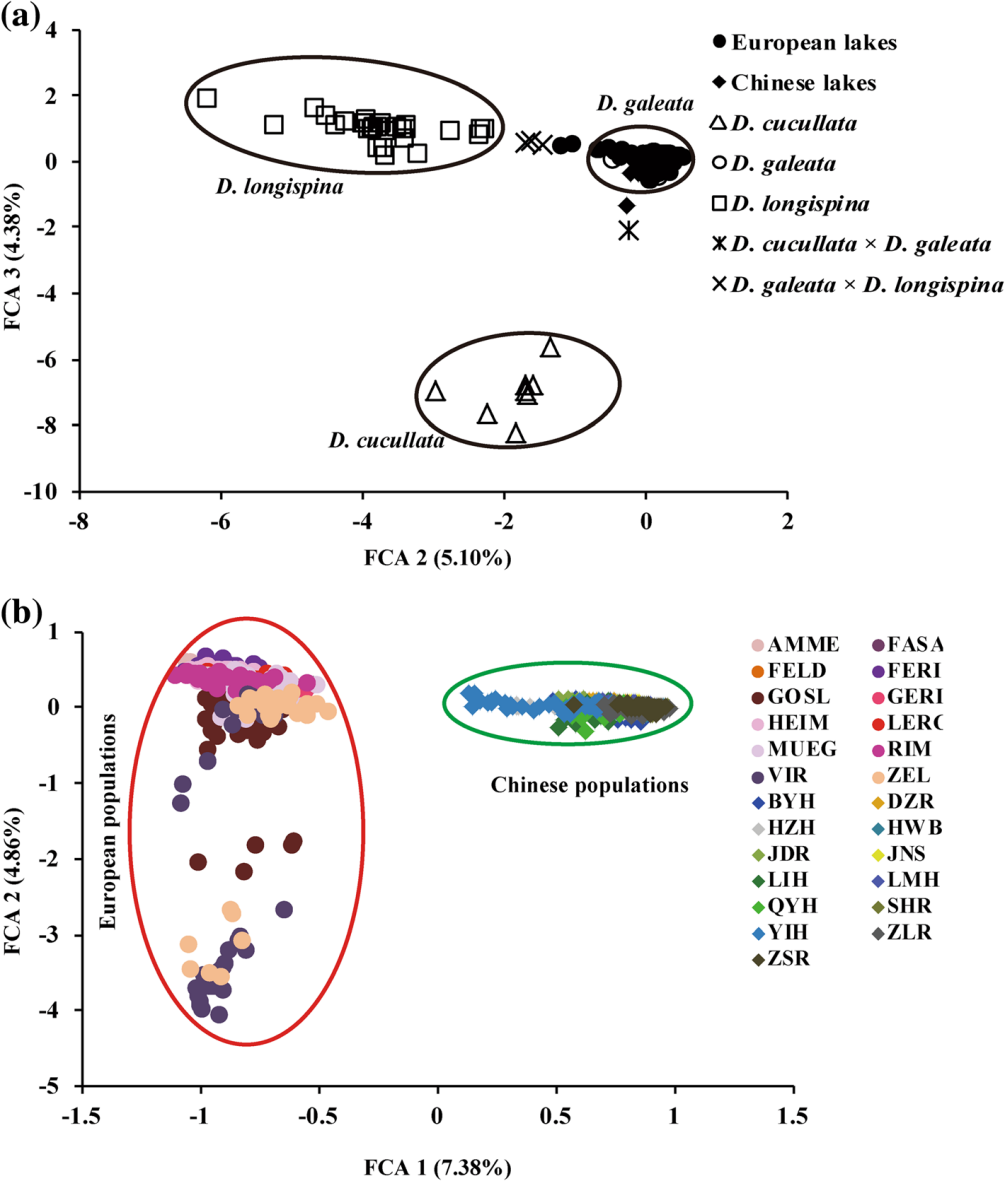
Results of the Factorial Correspondence Analysis (FCA) based on allelic variation at up to 15 microsatellite loci. **a** Species assignment of *Daphnia* individuals from Eastern China and Central Europe. Reference genotypes representing three species of the *D. longispina* complex and their interspecific hybrids (indicated by crosses) are included (for a list of all reference genotypes, see [20]). **b** Genetic similarities among pure *D. galeata* individuals sampled from Eastern China and Central Europe. For lake abbreviations see Table 1. Note that analyses in (**a**) and (**b**) are independent, and thus the axes are not comparable

**Fig. 3:**
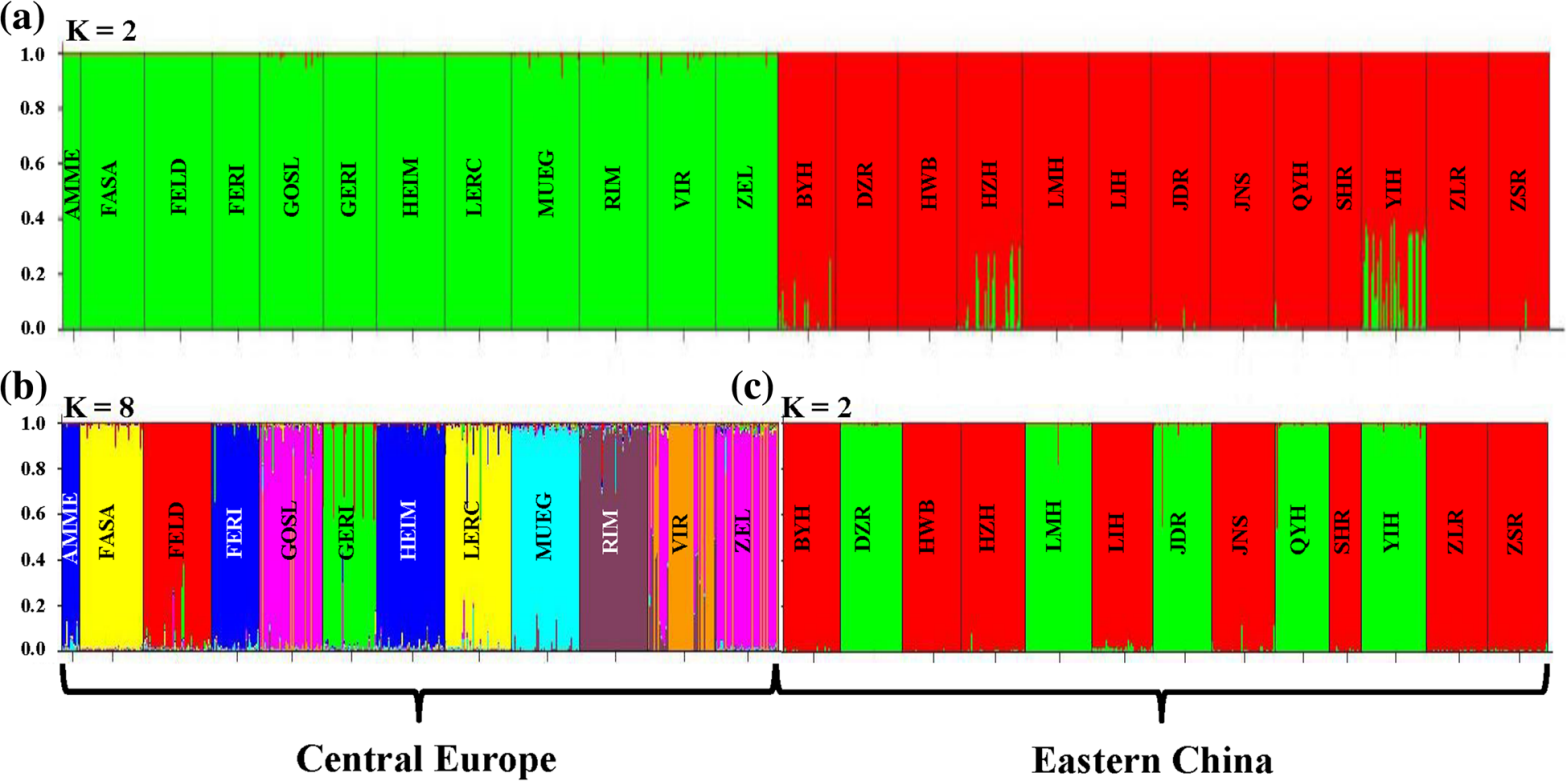
Results from a Bayesian assignment analysis (STRUCTURE) of microsatellite data for: **a** all 25 *D. galeata* populations, **b** 12 European populations, and **c** 13 Chinese populations. The best *K* is equal to **a** 2, **b** 8 and **c** 2, according to the method reported in [33]. The assignment of each individual to the respective groups is given. For lake abbreviations see Table 1

Based on polymorphism at 15 microsatellite loci, all 521 *Daphnia* individuals from Eastern China and 493 individuals from Central Europe samples clustered together with reference *D. galeata* genotypes in the FCA plot (Fig. 2a). Based on NewHybrids, 1004 out of 1014 individuals were assigned to pure *D. galeata* (with 90% posterior probability). FCA of these *D. galeata* individuals (without reference genotypes) revealed two main clusters clearly separating Chinese and European populations (Fig. 2b). There was no further separation within the Chinese cluster. However, some *D. galeata* individuals from European GOSL, VIR and ZEL populations were distinct from individuals from other European populations. Assignment test in STRUCTURE run on the entire *D. galeata* dataset showed that the most appropriate estimate of groups was *K* = 2, separating Chinese and European populations (Fig. 3a). In separate STRUCTURE analyses for Chinese and European populations, the most appropriate estimate of groups was *K* = 2 and *K* = 8, respectively (Fig. 3b and c). A UPGMA tree based on genetic distances confirmed that the Chinese samples were divergent from the European ones (Additional file 2: Figure S1). The observed UPGMA clustering within regions was to a large extent consistent with the assignment by STRUCTURE, especially for Chinese populations. Specifically, both methods revealed two clusters of Chinese populations; however, LMH and LIH populations were inconsistently assigned between these two clusters (compare Fig. 3c and Additional file 2: Figure S1).

**Fig. 4:**
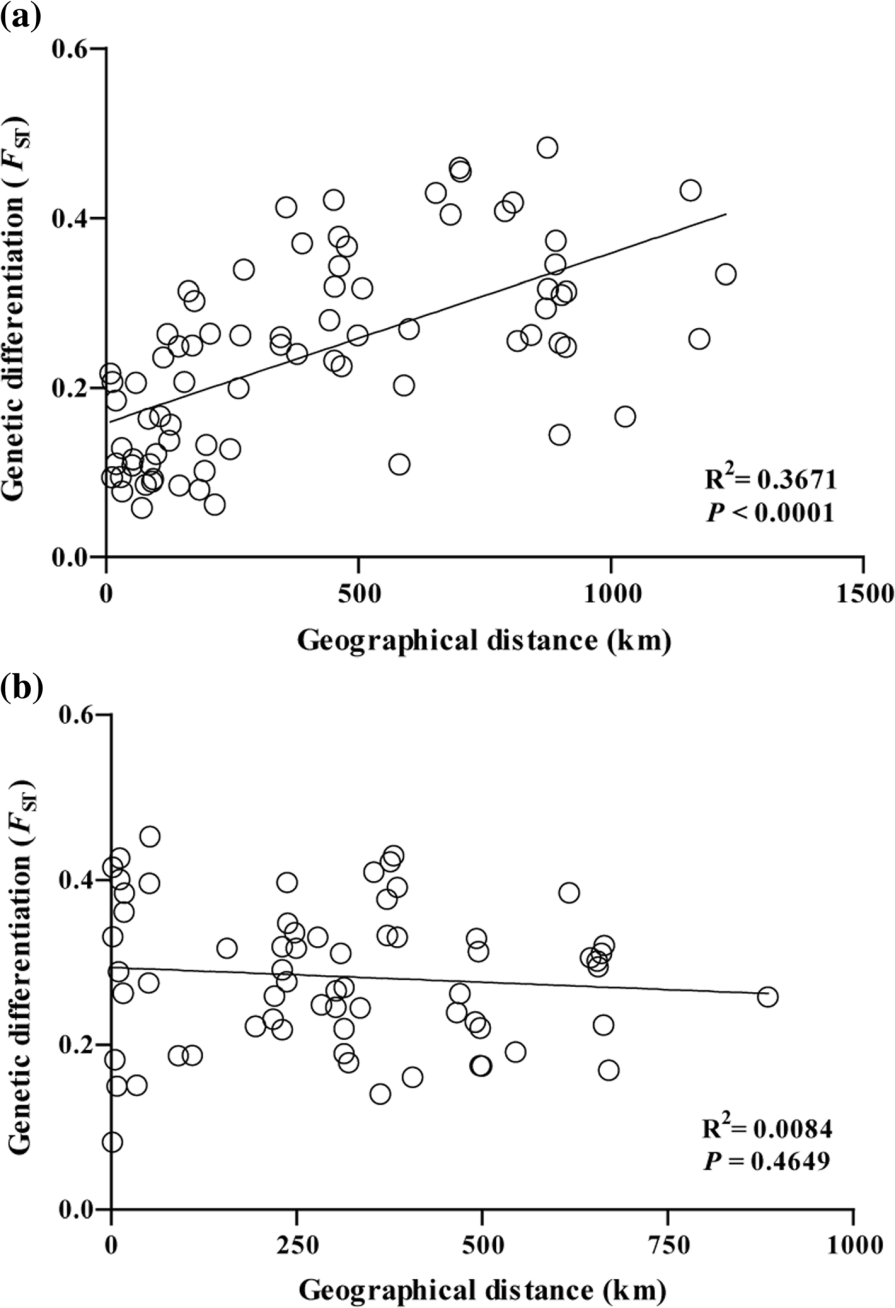
Scatterplot of pairwise geographical distance (kilometres) versus genetic distance (*F*_*ST*_ based on up to 15 microsatellite loci) among *D. galeata* populations from **a** Eastern China and **b** Central Europe

The AMOVA showed that most of the variation in the microsatellite dataset was explained by a within-population component (48%) while another significant component was between regions (33%), followed by within-region variation (19%; Table 2). Pairwise *F*_*ST*_ values ranged from 0.06 to 0.48 among Chinese populations and 0.08 to 0.45 among European populations (averaged over all loci). There was evidence of genetic isolation by distance for Chinese *D. galeata* populations (this remained significant even when two geographically distinct populations, YIH and DZR, were removed; data not shown), but not for European ones (Fig. 4a and b). Neither relative clonal richness nor allelic richness (Table 1) differed between the sets of Chinese and European populations (*t* = 2.06, *p* = 0.99 and *t* = 0.37, *P* = 0.53, respectively). No MLGs were shared among populations on continental or on local scales.

**Table 2:** Hierarchical analysis of molecular variance (AMOVA) for *D. galeata* populations, based on microsatellites and mtDNA, respectively. Between-region (i.e. Eastern China and Central Europe) variation is estimated in relation to within-region and within-population components

Marker type	Source of variation	DF	Explained variation (%)	*P*
Microsatellites	Between regions	1	32.80	< 0.001
	Among populations (within region)	23	18.91	< 0.001
	Among individuals (within population)	1983	48.29	< 0.001
mtDNA	Between regions	1	71.67	< 0.005
	Among populations (within region)	22	12.62	< 0.001
	Among individuals (within population)	230	15.71	< 0.001

### Mitochondrial DNA (12S gene)

**Fig. 5:**
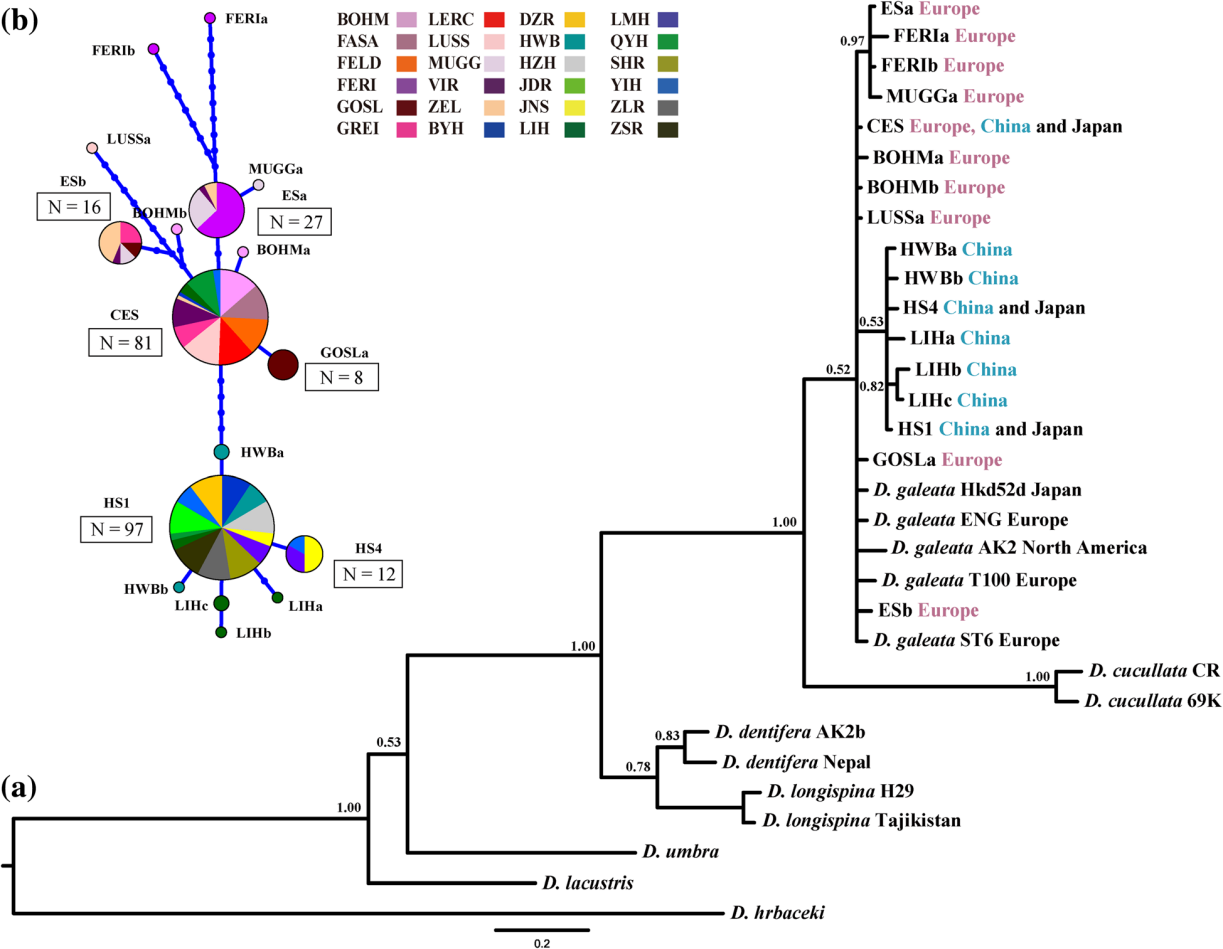
**a** Bayesian phylogenetic tree and **b** haplotype network of *D. galeata*, based on the variation of the mitochondrial 12S rRNA gene (430 bp). Codes of *Daphnia* individuals are provided in Table 1; for origin of reference sequences see Additional file 1: Table S1. Less than 70% support of nodes is not shown. *Daphnia hrbaceki* was used as an outgroup. Each circle in **b** represents a unique haplotype, and its size reflects the number of individuals carrying that particular haplotype. Segment sizes within circles correlate with the distribution of haplotypes among different populations. Colour codes allow easy discrimination between European populations (purple to red) and Chinese ones (blue, green, grey) in the network. For lake abbreviations see Table 1

Two hundred fifty-four individuals analysed at the 12S rRNA locus (430 bp in the aligned dataset) were assigned to *D. galeata* (Fig. 5); among these, 17 unique haplotypes were detected (Table 1). The level of haplotype diversity or nucleotide polymorphism within analysed *D. galeata* lineages did not differ between Chinese and European populations (*t* = 0.07, *P* = 0.26 and *t* = 0.31, *P* = 0.14, respectively; Table 1). Generally, there was a separation between Chinese and European haplotypes, which was confirmed by both Bayesian inference of phylogeny and network analysis (Fig. 5a and b); however, one widespread haplotype (CES) was shared among China, Japan and Europe. Two additional haplotypes (HS1, HS4) were shared between China and Japan (Fig. 5a). Shared haplotypes were also observed within the studied regions: three haplotypes (CES, HS1 and HS4) were shared among populations from China, and three (CES, ESa and ESb) among populations from Europe (Fig. 5b, Table 1). An AMOVA test revealed that about six times more genetic variation was attributable to between-region than within-region (i.e. among-population), or within-population (i.e. among-individuals) levels (Table 2).

## Discussion

In the present study, we compared the patterns of genetic variation in geographically distant populations of a widespread *Daphnia* species, *D. galeata*. Specifically, multiple populations of *D. galeata*, sampled from two remote geographical regions (Eastern China and Central Europe), were screened using mitochondrial (12S) and nuclear markers (microsatellites). One of seventeen mitochondrial haplotypes described here was shared between European and Chinese populations. This haplotype (CES) was also widespread within both regions, having been detected in eight out of eleven studied European populations and four out of thirteen Chinese populations. The network analysis (Fig. 5b) demonstrates that this haplotype is closely related to other European haplotypes, and thus likely has a Western Palaearctic origin. Another abundant haplotype, HS1, was detected in all thirteen Chinese habitats, but not in European ones; this might be a founding mtDNA lineage for Chinese haplotypes. The third abundant haplotype, ESa, was shared between four European populations. Notably, the CES and ESa haplotypes were also detected in a previous study of European populations of the *D. longispina* complex [47]. There, the CES haplotype was present in *D. galeata* populations from the Netherlands and Czechia, and the ESa haplotype in populations from the Netherlands.

Based on microsatellite markers, a substantial separation was observed not only between Chinese and European populations, but also within Europe, between three *D. galeata* populations (two Czech: VIR and ZEL, and one Polish: GOSL) and nine remaining populations. The separation of these three populations was well supported by FCA, clustering method based on genetic distances (in Bayesian assignment, some individuals from VIR and most from GOSL and ZEL clustered together) and UPGMA analysis. The clear genetic separation between Chinese and European populations could be explained by the large geographical isolation between these two parts of the Palaearctic. However, the geographical position of localities of *D. galeata* populations within Europe was not associated with their genetic similarity, suggesting gene flow occurs within regions. A genetic separation of VIR, ZEL and GOSL from other *D. galeata* populations in Europe might have resulted from introgression of *D. cucullata* to *D. galeata*. In all three localities, we detected *D. cucullata* (although its hybrids with *D. galeata* apparently were not very common in ZEL and VIR [28]), whereas communities of other analysed European lakes often consist of *D. galeata* hybridizing with *D. longispina* [24, 48, 49]. Furthermore, we observed a discordant pattern between mitochondrial and nuclear markers, as some individuals from the above localities were classified to *D. galeata* based on microsatellites, but carried *D. cucullata* mitochondrial haplotypes (data not shown). This suggests that the introgression of the mitochondrial genome occurs after initial hybridization, as observed in other animals (e.g. [50, 51]).

Interestingly, there was a significant association between genetic distance and geographical distance for *D. galeata* populations from China (but not from Europe). One explanation for the discrepancy could be that studied European *D. galeata* habitats are ecologically more variable than the mostly eutrophic Chinese lakes [52]. In addition to isolation by distance, ecological differences among habitats (i.e., isolation by environment), such as differences in trophic level, could also affect the genetic composition of the *D. longispina* complex assemblages [14]. Because of such selective constraints, a pattern of isolation by distance will not always be the rule. Our findings are in line with previous studies on the *D. longispina* complex where mixed results were reported. Specifically, a significant association between genetic distance (measured by allozyme markers) and geographical distance was detected for other taxa of the *D. longispina* complex [53] but in a large-scale study of *D. longispina*, no signal for genetic isolation by distance was observed [47].

In general, most of the *D. galeata* populations investigated here, regardless of their origin, showed high relative clonal richness values (as assessed by microsatellites), likely a result of frequent sexual reproduction, which recreates genotypic variation. High clonal richness of *D. galeata* populations was also observed previously (e.g. [47]). However, frequent sexual reproduction in *D. galeata* populations is not always the case; a substantially lower clonal variation in *D. galeata* populations was observed in high mountain lakes where populations overwinter under ice, accompanied by a prolonged period of clonal selection [54]. Many populations analysed by us, especially the Chinese ones that are in localities with a mild climate [55], may survive all year round. However, despite possible overwintering by a fraction of the population, these populations also show substantial clonal richness. This might be caused by external conditions promoting genetic polymorphism, such as coevolving parasites [56, 57].

## Conclusions

A high number of polymorphic nuclear loci and their codominant inheritance often provide fine resolution in detecting genetic structure [23]. Here, based on a set of high resolving microsatellite loci, *D. galeata* populations from two regions, i.e., Eastern China and Central Europe, were found to be clearly separated from each other, which was confirmed by three types of analyses (FCA, Bayesian assignment and clustering analysis based on genetic distances). This principal finding was supported by mitochondrial DNA data; however, we detected in both regions a common and widespread mtDNA haplotype that was shared across the whole Palaearctic. Our finding calls for further studies to investigate the colonization history of *D. galeata*, and zooplankton in general, in China and adjacent regions.

## Additional files

Additional file 1: **Table S1.** Reference sequences of the *D. longispina* complex used in phylogenetic analyses. (DOCX 15 kb)

Additional file 2: **Figure S1**. UPGMA clustering of *D. galeata* populations sampled from Eastern China and Central Europe based on microsatellite polymorphism at 15 loci. For lake abbreviations see Table 1. (TIF 4103 kb)
