# Supplementary material for: Cytonuclear diversity and shared mitochondrial haplotypes among Daphnia galeata populations separated by seven thousand kilometres
Source: BMC Evol Biol. 2018 Sep 3;18:130. doi: 10.1186/s12862-018-1256-4 (PMC6122193; doi:10.1186/s12862-018-1256-4)
Supplement: Supplementary file 1 — Table S1. Reference sequences of the D. longispina complex used in phylogenetic analyses. (DOCX 15 kb) [file 12862_2018_1256_MOESM1_ESM.docx]

Table S1. Reference sequences of the *D. longispina* complex used in phylogenetic analyses.

| Species | ID | Location | 12S GenBank | References |
| --- | --- | --- | --- | --- |
|  |  |  | Accession No. |  |
| *D. galeata* | HS4 | A pond in Raihukudai, Yamaguchi; Japan | AB642042 | Ishida et al. 2011, BMC Evol. Biol. |
| *D. galeata* | HS1 | Oginoike Pond, Hiroshima; Japan | AB641994 | Ishida et al. 2011, BMC Evol. Biol. |
| *D. galeata* | CES | Lake Kawaguchi, Yamanashi; Japan | AB642048 | Ishida et al. 2011, BMC Evol. Biol. |
| *D. galeata* | T100 | Lake Tjeukemeer; Netherlands | EF375851 | Petrusek et al. 2008, Zool. Scr. |
| *D. galeata* | AK2 | Mirror Lake, AK; USA | AY730365 | Taylor et al. 2005, Mol. Ecol. |
| *D. galeata* | ENG | Ullswater; England | AY730360 | Taylor et al. 2005, Mol. Ecol. |
| *D. galeata* | Hkd52d | Lake Shikotsu, Hokkaido; Japan | AB642002 | Ishida et al. 2011, BMC Evol. Biol. |
| *D. galeata* | ST6 | Stanovice; Czechia | FJ178305 | Thielsch et al. 2009, Mol. Ecol. |
| *D. cucullata* | CR | Medlov Pond; Czechia | AF277270 | Schwenk et al. 2000, Proc. R. Soc. Lond., B, Biol. Sci. |
| *D. cucullata* | 69K | Lake Akersvann; Norway | EF375854 | Petrusek et al. 2008, Zool. Scr. |
| *D. dentifera* | AK2b | Teller6 Pond, AK; USA | AY730374 | Taylor et al. 2005, Mol. Ecol. |
| *D. dentifera* | Nepal | Khumbu region; Nepal | JX446618 | Moest et al. 2012, Hydrobiologia |
| *D. longispina* | H29 | Lake Constance; Germany | EF375829 | Petrusek et al. 2008, Zool. Scr. |
| *D. longispina* | Tajikistan | Lake Rangkul, Pamir Mountains; Tajikistan | JX446619 | Moest et al. 2012, Hydrobiologia |
| *D. lacustris* |  | Lake Maridalsvann; Norway | DQ337943 | Nilssen et al. 2007, Hydrobiologia |
| *D. 'umbra'* |  | Pond Mallalampi A; Finland | EF375849 | Petrusek et al. 2008, Zool. Scr. |
| *D. hrbaceki* |  | Cesky prikop near Nosalov, Kokorinsko region; Czechia | HM625747 | Juracka et al. 2010, Zootaxa |
