# Supplementary figures and images for: Cytonuclear diversity and shared mitochondrial haplotypes among Daphnia galeata populations separated by seven thousand kilometres
Source: BMC Evol Biol. 2018 Sep 3;18:130. doi: 10.1186/s12862-018-1256-4 (PMC6122193; doi:10.1186/s12862-018-1256-4)

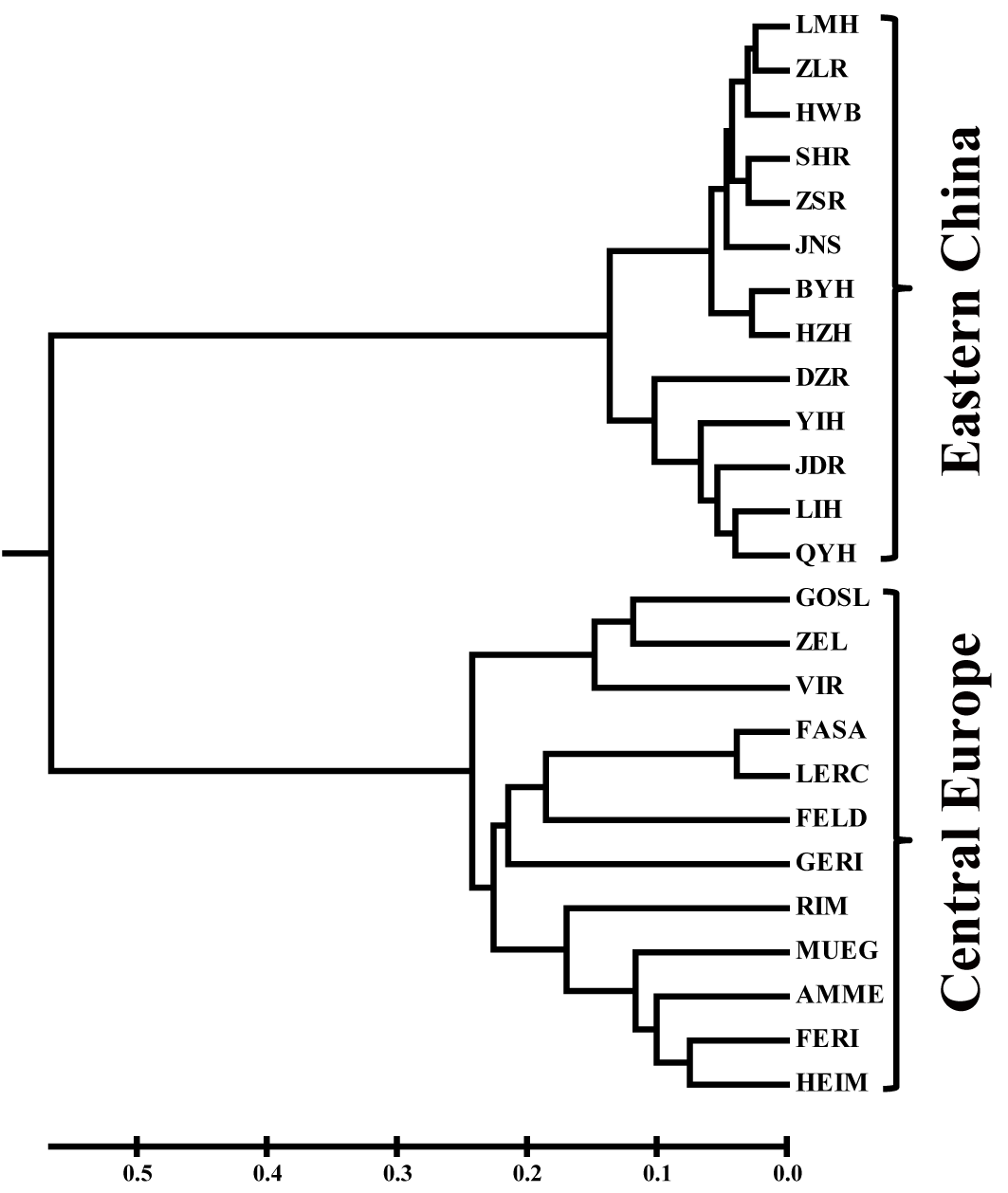

Supplement: Supplementary file 2 — Figure S1. UPGMA clustering of D. galeata populations sampled from Eastern China and Central Europe based on microsatellite polymorphism at 15 loci. For lake abbreviations see Table 1. (TIF 4103 kb) [file 12862_2018_1256_MOESM2_ESM.tif]
